# Heterogeneity of tumor immune microenvironment and real-world analysis of immunotherapy efficacy in lung adenosquamous carcinoma

**DOI:** 10.3389/fimmu.2022.944812

**Published:** 2022-08-12

**Authors:** Chao Li, Xiaobin Zheng, Pansong Li, Huijuan Wang, Jie Hu, Lin Wu, Zhijie Wang, Hui Guo, Fang Wu, Wenzhao Zhong, Chengzhi Zhou, Qian Chu, Jun Zhao, Xinlong Zheng, Weijin Xiao, Weifeng Zhu, Longfeng Zhang, Qian Li, Kan Jiang, Qian Miao, Biao Wu, Yiquan Xu, Shiwen Wu, Haibo Wang, Shanshan Yang, Yujing Li, Xuefeng Xia, Xin Yi, Cheng Huang, Bo Zhu, Gen Lin

**Affiliations:** ^1^ Department of Pathology, College of Clinical Medicine for Oncology, Fujian Medical University, Fujian Cancer Hospital, Fuzhou, China; ^2^ Fujian Key Laboratory of Translational Cancer Medicine, Fuzhou, China; ^3^ Department of Thoracic Oncology, College of Clinical Medicine for Oncology, Fujian Medical University, Fujian Cancer Hospital, Fuzhou, China; ^4^ Geneplus-Beijing Institute, Beijing, China; ^5^ Henan Cancer Hospital/Affiliated Cancer Hospital of Zhengzhou University, Zhengzhou, China; ^6^ Department of Pulmonary Medicine, Shanghai Respiratory Research Institute, Zhongshan Hospital Affiliated to Fudan University, Shanghai, China; ^7^ Department of Thoracic Medical Oncology, The Affiliated Cancer Hospital of Xiangya School of Medicine, Central South University/Hunan Cancer Hospital, Changsha, China; ^8^ State Key Laboratory of Molecular Oncology, Department of Medical Oncology, National Cancer Center/Cancer Hospital, Chinese Academy of Medical Sciences and Peking Union Medical College, Beijing, China; ^9^ Department of Medical Oncology, The First Affiliated Hospital of Xi’an Jiaotong University, Xi’an, China; ^10^ Department of Oncology, The Second Xiangya Hospital of Central South University, Changsha, China; ^11^ Guangdong Lung Cancer Institute, Guangdong General Hospital and Guangdong Academy of Medical Sciences, Guangzhou, China; ^12^ The First Affiliate Hospital of Guangzhou Medical University, Guangzhou, China; ^13^ Department of Oncology, Tongji Hospital of Tongji Medical College, Huazhong University of Science and Technology, Wuhan, China; ^14^ Key Laboratory of Carcinogenesis and Translational Research (Ministry of Education), Department of Thoracic Medical Oncology‐I, Peking University Cancer Hospital and Institute, Beijing, China; ^15^ Chongqing Key Laboratory of Immunotherapy, Chongqing, China; ^16^ Institute of Cancer, Xinqiao Hospital, Third Military Medical University, Chongqing, China

**Keywords:** lung adenosquamous carcinoma, tumor immune microenvironment, heterogeneity, PD-L1 expression, immune checkpoint inhibitor

## Abstract

Lung adenosquamous carcinoma (ASC) is an uncommon histological subtype. We aimed to characterize the tumor immune microenvironment (TIME) in lung ASC and estimate patient response to immune checkpoint inhibitors (ICIs), which have never been systematically investigated. In cohort I, we collected 30 ASCs from a single center for analysis of TIME characteristics, including immuno-phenotyping, tumor mutation burden (TMB), T-cell receptor (TCR) repertoires, tumor-infiltrating lymphocytes (TILs), and immune checkpoint expression. Twenty-two (73.3%) patients were EGFR-positive. The TIME was defined by immune-excluded (60%) and immune-desert phenotype (40%). Strikingly, programmed cell death-ligand 1 (PD-L1) and programmed cell death-1 (PD-1) were predominantly expressed in squamous cell carcinoma components (SCCCs) versus adenocarcinoma components (ACCs), where enhanced CD4^+^ FOXP3^+^ regulatory T cell and attenuated CD57^+^ natural killer cell infiltration were present, consistent with a landscape of fewer innate immune cells, more immunosuppressive cells. SCCCs had higher TMB, higher TCR clonality, and lower TCR diversity than ACC. In cohort III, the efficacy of ICI-based therapy was estimated using a real-world data of 46 ASCs from 11 centers. Majority of 46 patients were driver genes negative and unknown mutation status, 18 (39%) and 18 (39%), respectively. The overall objective response rate of 28%, median progression-free survival of 6.0 months (95% confidence interval [CI] 4.3–7.7), and median overall survival of 24.7 months (95% CI 7.2–42.2) were observed in the ICI-based treatment. This work ascertains suppressive TIME in lung ASC and genetic and immuno-heterogeneity between ACCs and SCCCs. Lung ASC patients have a moderate response to ICI-based immunotherapy.

## Introduction

Lung adenosquamous carcinoma (ASC) is an uncommon histological subtype, accounting for 0.4% to 4% of lung cancers (1, 2). Pathologically, ASC consists of adenocarcinoma components (ACCs) and squamous cell carcinoma components (SCCCs), according to the World Health Organization (WHO) histologic classification of lung cancers (3). Due to the absence of specific management for lung ASC, current options are confined to those listed in non-small cell lung cancer (NSCLC) guidelines. However, it is pathologically heterogeneous and thereby widely accepted as an NSCLC subtype more difficult to treat than classical lung adenocarcinoma (LUAD) and squamous cell carcinoma (LUSC), often associated with a worse prognosis (4–6).

Recently, ACCs and SCCCs of ASC have been proven to stem from a monoclonal cell ancestor and evolve into two subtypes. A plausible theory for the monoclonal origin of ASC that receives from the evidence of shared driver genes in ACC and SCCC, such as mutated *EGFR* and (Kirsten ras) *KRAS* genes, has been more appreciated (7–10). Our recent study of genomic profiling of paired ACCs and SCCCs using a 1,021-gene panel has provided another piece of substantial evidence to support the theory (11). Specifically, the two types of pathological components have trunk alterations in the phylogenetic tree, *EGFR*, *TP53*, *ERBB2*, *PIK3CA* mutations, and *EGFR* copy number gain and *MDM2* copy number loss in the trunk. The high frequency of *EGFR* mutations and similarity between genomic landscapes of lung ASC trunk mutations and pure LUAD indicate that lung ASC may originate from a subset of glandular cancer cells. ACCs and SCCCs are genetically heterogeneous due to universal branch evolution. ASC patients harboring *EGFR* mutations may benefit from EGFR tyrosine kinase inhibitors (EGFR-TKIs). All these studies have offered improved insight into ASC origin for better treatment options.

Immune checkpoint inhibitor (ICI)–based therapies targeting CTLA4 or PD1/PD-L1 have achieved impressive success in the treatment of lung cancer over the last decade. Mechanisms of how the complex tumor immune microenvironment (TIME) determines response to ICI therapies have been highlighted (12). However, whether lung ASC patients can benefit from immunotherapy remains uncertain due to the scarce knowledge or clinical data on the TIME of the rare subtype. Shi et al. reported comparable PD-L1 expression between lung ACCs and LUAD (11.1% and 13.5%), lung SCCCs and LUSC (38.89% and 28.9%) (13). That means anti-PD1/L1 agents can be effective for certain lung ASC cases. Thus, a more comprehensive understanding of ASC TIME for the development of disease management is needed.

In this study, our objectives are twofolded: to characterize the heterogeneity of the TIME within lung ASC and estimate patient response to ICIs. We retrospectively analyzed the genetic and clinicopathological data from lung ASC patients for assessments of immunophenotype, lymphocyte infiltration pattern, immune checkpoint expression, tumor mutation burden (TMB), and T-cell receptor (TCR) repertoire and the estimation of immunotherapy efficacy. This work will offer a fundamental understanding of TIME patterns closely associated with ICI-based treatments for lung ASC.

## Methods

### Patients and samples

Three independent cohorts of patients were enrolled. Cohort I, for assessment of immune parameters, contained 30 primary tumor samples from consecutive patients who underwent complete resection at Fujian Cancer Hospital between June 2011 and December 2018 but did not receive any anticancer treatment prior to surgery. Two board-certified pathologists independently reviewed all samples and performed immunohistochemistry (IHC). IHC biomarkers including TTF-1, Napsin A, p40, and CK5/6 were utilized to identify and distinguish ACCs and SCCCs (11). Cohort II of 60 LUAD and LUSC from Geneplus-Beijing (https://www.geneplus.org.cn/) were selected for TMB and TCR comparisons. In Cohort III, 46 lung ASC patients with complete efficacy and survival data from 11 cancer centers were included for estimation of immunotherapy efficacy based on the following conditions: (1) patients who were diagnosed with stage IV lung ASC or suffered a relapse after surgery, (2) and treated with anti–PD-1/PD-L1 agents (single or combined with chemotherapy) for at least one cycle, (3) and received efficacy evaluation per RECIST v1.1 criterion at least once after the first dose (baseline).

### Microdissection of tumor samples

IHC biomarkers, namely, TTF-1, NapsinA, p40, and CK5/6 were used to distinguish and identify ACCs and SCCCs. Manual microdissection was carried out in the area where the ACCs and SCCCs are clearly separated. Laser-capture microdissection was carried out on selective samples that contained both histologic components that are not separable by normal manual microdissection.

### Immune cell phenotyping

Immune cell phenotyping was performed based on the density and localization of lymphocytes, as previously described (14). Briefly, the density and localization of lymphocytes were determined on hematoxylin and eosin (H&E) stained sections per recommendations in the International TILs Working Group 2014 (15). The tumor-infiltrating lymphocyte (TIL) landscape of all mononuclear cells from patients was assessed in the stromal compartment within the borders of an invasive lesion at five random fields in each section at ×200 magnification. The proportion of each TIL subpopulation was calculated based on the percentage of the area occupied by mononuclear cells over the stromal area both around the tumor border and inside the tumor mass. The mean TIL percentage of each sample was recorded. Patients were classified into three groups: (1) immune-inflamed phenotype, depending on the lymphocyte density detected inside the tumor mass in proximity to tumor cells was ≥ 10%, regardless of lymphocyte status in the stromal area around the tumor border; (2) immune-excluded, with the lymphocyte density in the stromal area around the tumor border of ≥10% and a negligible (<10%) amount of lymphocytes inside the tumor mass; (3) immune-desert phenotype, wherein the lymphocyte density was negligible (<10%) in both the tumor mass and the stromal area.

### Next-generation sequencing and tumor mutation burden estimation

DNA and sequencing libraries were prepared using a sequencing panel of 1,021 cancer-related genes, as described in our previous report (11). Libraries were sequenced to a uniform median coverage of 515×. Somatic mutations with a variant allele fraction (VAF) ≥2% and at least five high-quality reads (a Phred score ≥30, mapping quality ≥30, and without paired-end read bias) were identified. TMB was defined as the number of all nonsynonymous mutations per 0.7 Mb of targeted coding regions.

### T-cell receptor sequencing

FFPE DNA was amplified in a bias-controlled multiplex polymerase chain reaction (PCR) system. Then, we performed human TCRβ chain complementarity-determining region 3 (CDR3) profiling through high-throughput sequencing on the IR-seq platform (Geneplus-Beijing, Beijing, China) (16). CDR3 sequences were identified and assigned using the MiXCR software package (17). TCRβ CDR3 diversity of lung ASC tissues was calculated based on the Shannon entropy index, which is a function of both the relative number of clonotypes present and the relative abundance or distribution of each clonotype (16). TCR clonality was employed to assess the clonal expansion of tumor-specific T cells and defined as 1– (Shannon entropy index)/ln(number of productive unique sequences) (18).

### Multiple immunofluorescences staining

Multiple immunofluorescences (MIFs) staining was performed on sections (4-mm thickness) from FFPE human lung ASC samples derived from cohort I patients. All slides were deparaffinized manually using xylene, rehydrated in a graded ethanol series, and washed in tap water before microwave treatment (MWT) for heat-induced epitope retrieval in tris-EDTA buffer (pH 9; 643901; Klinipath, Duiven, the Netherlands). Endogenous peroxidase was blocked with Antibody Diluent/Block (72424205; PerkinElmer, Massachusetts, USA). Protein blocking was performed using Antibody Diluent/Block. One antigen requires one round of labeling, including primary antibody incubation, secondary antibody incubation, and tyramide signal amplification (TSA) visualization, followed by labeling of the next antibody.

Two panels were used to perform multiple immunofluorescence (MIF) staining. CD3 (ZM0417, Zsbio Beijing, China), CD4 (ZM0418, Zsbio Beijing, China), FOXP3 (ab20034, Abcam Cambridge, UK), TIM3 (CST45208S, Cell Signaling Technology Massachusetts, USA), and LAG3 (ab40468, Abcam Cambridge, UK) were tested in panel 1, whereas CD8 (ZA0508, Zsbio Beijing, China), CD57 (ZM0058, Zsbio Beijing, China), CD68 (ZM0060, Zsbio Beijing, China), CD163 (ZM0428, Zsbio Beijing, China), PD1 (ZM0381, Zsbio Beijing, China), and PD-L1 (ZA0629, Zsbio Beijing, China) in panel 2. Primary antibodies CD3, CD57, CD68, PD-1, PD-L1, LAG3, TIM3, and FOXP3 were applied on slides for 1-h incubation at 37°C, and anti-CD4, CD8, and CD163 antibodies were employed for overnight incubation at 4°C, followed incubation with Opal Polymer HRP Ms+Rb (2414515; PerkinElmer, Massachusetts, USA) for 10 min at 37°C. TSA visualization was performed with the Opal seven-color IHC Kit (NEL797B001KT; PerkinElmer, Massachusetts, USA), containing fluorophores 4′,6-diamidino-2-phenylindole (DAPI), Opal 520 (CD3), Opal 570 (CD4), Opal 620 (LAG3), Opal 650 (TIM3), Opal 690 (FOXP3), Opal 620 (CD57), Opal 520 (CD68), Opal 570 (PD-L1), Opal 540 (CD80, Opal 650 (PD1), Opal 690 (CD163), and TSA Coumarin system (NEL703001KT; PerkinElmer, Massachusetts, USA). MWT was performed with tris-EDTA buffer (pH 9) to remove antibody-TSA complexes. TSA single-stained slides were made with MWT, counterstained with DAPI for 5 min, and mounted with Antifade Mounting Medium (I0052; NobleRyder, Beijing, China).

### Tissue imaging and MIF data analysis

Slides were scanned for electronic review using the PerkinElmer Vectra (Vectra 3.0.5; PerkinElmer, Massachusetts, USA). Multispectral images were unmixed using spectral libraries built from images of the library stains for each fluorophore using the inForm Advanced Image Analysis software (inForm 2.3.0; PerkinElmer, Massachusetts, USA). A selection of five to 10 representative original multispectral images was utilized for training of the inForm Tissue Finder to learn tissue and cell segmentation, identify phenotypes, and estimate positivity scores. All the settings applied to the training images were saved within an algorithm to allow a batch analysis of multiple original multispectral images of the same sample (19). TIL infiltration pattern and type in tumorous, stromal, and total regions (the sum of tumorous and stromal areas) was investigated.

### PD-L1 immunohistochemistry

PD-L1 expression was verified using the Dako PD-L1 IHC 22C3 pharmDx assay (Agilent Technologies, Santa Clara, CA, USA). The tumor proportion score (TPS) of PD-L1 was recorded as the percentage of at least 100 viable tumor cells exhibiting complete or partial PD-L1 membrane staining (20). Pathologists from a certified commercial vendor provided TPS interpretations.

### Efficacy and outcome measures

The best response of complete response (CR), partial response (PR), stable disease (SD), and progressed disease (PD) after immunotherapy (single or combined with chemotherapy) were recorded. Objective response rate (ORR) and disease control rate (DCR) were calculated. ORR was defined as the proportion of patients achieving CR and PR, and DCR the proportion of those achieving CR, PR, and SD. Progression-free survival (PFS) was recorded from initiation of treatment with immunotherapy until disease progression, death from any cause, or the last follow-up visit. Overall survival (OS) was measured from treatment initiation to death.

### Statistical analysis

All data were analyzed using R Package (Version 3.3.0) or Prism 5.0 (GraphPad Software Inc., La Jolla, CA, USA). The Mann–Whitney test was applied for unpaired observations and Wilcoxon matched-pairs signed-rank test for paired data. Spearman correlation was applied to assess the correlation of TMB with immune checkpoint expression, TIL infiltration, and TCR diversity. PFS and OS were illustrated using the Kaplan–Meier method. A two-sided *p* < 0.05 was considered statistically significant.

## Results

### Lung adenosquamous carcinoma shows immune-excluded and immune-desert immunophenotype

**Figure 1:**
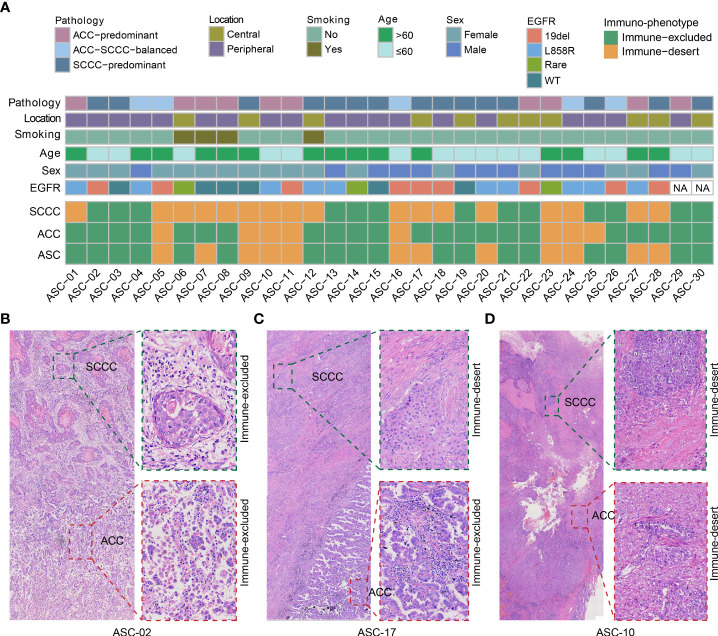
Patient characteristics and immunophenotypes of lung adenosquamous carcinoma (ASC). **(A)** Demographic and clinicopathological characteristics and immunophenotypes based on the density and localization of tumor-infiltrating lymphocytes. All adenocarcinoma component (ACC) and squamous cell carcinoma component (SCCC) samples show immune-excluded or immune-desert immunophenotypes. (**B–D**) Images of classical immunophenotypes.

Thirty patients in cohort I were recruited for immu-nophenotyping. Patient characteristics were summarized in **Figure 1A**. Pathologically, ACC-predominant, SCCC-predominant, and ACC-SCCC-balanced samples accounted for 33% (*n* = 10), 50% (*n* = 15), and 17% (*n* = 5). Mutationally, *EGFR* mutation status was tested in 28 patients, 22 of whom were *EGFR*-mutant (**Figure 1A**).

Immunophenotyping data of lung ASCs were analyzed by reviewing both ACC and SCCC regions on pathological images (**Figure 1A**). The results showed that immune-excluded and immune-desert phenotypes accounted for 60% and 40% of all ASCs, respectively. No immune-inflamed phenotype was found. Of lung ACC samples, 77% (23 out of 30) were immune-excluded, and the remanent immune-desert phenotype; 47% of SCCCs displayed immune-excluded versus 53% immune-desert. No immune-inflamed phenotype was found in either ACCs or SCCCs. About half of the patients had the same phenotype in ACCs and SCCCs, and the others unanimously showed immune-desert phenotype in SCCCs and immune-excluded in matched ACCs. Classical immunophenotypes were showed in **Figures 1B–D**.

### Predominant expression of PD-L1 in squamous cell carcinoma components versus adenocarcinoma components

**Figure 2:**
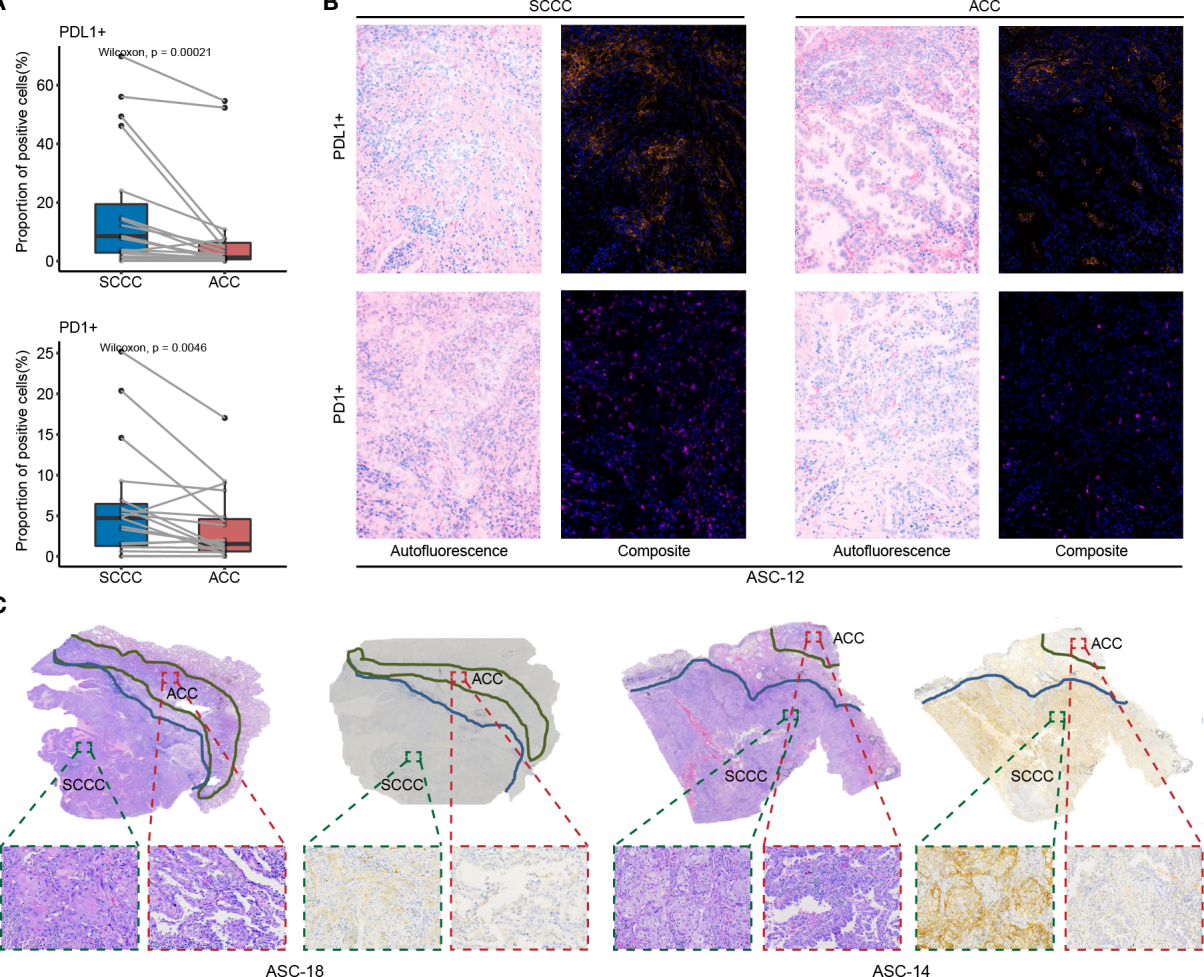
PD-L1 and PD-1 expression patterns in lung adenosquamous carcinoma (ASC). **(A)** PD-L1 and PD-1 expressions are assessed using multiple immunofluorescences. PD-L1 and PD-1 are strongly expressed in squamous cell carcinoma component (SCCCs) versus adenocarcinoma components (ACCs). **(B)** A case of classical ASC shows PD-L1 and PD-1 expressions in SCCCs and ACCs. **(C)** Two cases of classical ASC reveal PD-L1 and PD-1 expressions in SCCCs and ACCs. Dako PD-L1 immunohistochemistry shows the difference in PD-L1 expression between ACCs and SCCCs.

Expressions of four immune checkpoints, PD-1, PD-L1, TIM3, and LAG3, were quantified in paired ACCs and SCCCs using MIF assay (**Figure 2**). The density of positive cells was calculated in tumorous, stromal, and total regions (containing the tumor and stroma regions). Pronounced expressional heterogeneity of PD-L1 or PD-1 expression was detectable between SCCC and ACC in the three regions. SCCC illustrated strong and extensive PD-L1 expression (median proportion 8.5% vs. 1.2%, *p* < 0.001) and elevated PD-1 (4.7% vs. 1.5%, *p* = 0.0046) in the total region compared with ACCs (**Figure 2A**). One case of classic PD-L1/PD-1 expression was shown in **Figure 2B**. Similar results were observed in tumorous and stromal regions (**Supplementary Figures 1A, B**). The PD-L1 expression pattern was further supported by 22C3-based anti–PD-L1 antibody IHC (**Figure 2C**). Higher PD-L1/PD-1 expression was positively correlated with ACC and SCCC areas (**Supplementary Figures 2A, B**).

TIM3 and LAG3, representative PD-L1 bypass immune checkpoints, were identified with expression levels comparable in SCCCs and ACCs (**Supplementary Figure 1**). TIM3, other than LAG3, displayed positive correlations with SCCC and ACC areas in tumorous, stromal, and total regions, influenced by an anomalous value from one patient (**Supplementary Figures 2C, D**).

### Enhanced regulatory T cell and impaired natural killer cell infiltration in squamous cell carcinoma components indicate immunosuppressive phenotype

**Figure 3:**
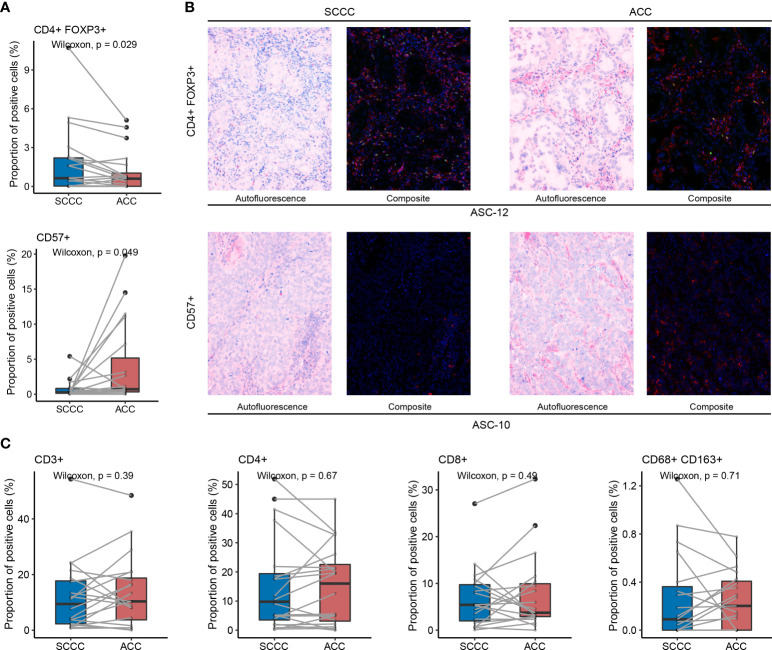
Tumor-infiltrating lymphocytes (TILs) status in lung adenosquamous carcinoma (ASC) evaluated by multiple immunofluorescences. **(A)** CD4^+^ FOXP3^+^ T_reg_ and CD57^+^ natural killer (NK) cell infiltration is reflected by their proportions in the total region of squamous cell carcinoma components (SCCCs) and adenocarcinoma components (ACCs). SCCCs reveal enhanced CD4^+^ FOXP3^+^ T_reg_ and impaired CD57^+^ NK cell infiltration compared with ACCs. **(B)** One case of typical CD4^+^ (green) FOXP3^+^ (red) T_reg_ infiltration and another of typical CD57^+^ NK cell infiltration using multiple immunofluorescences. **(C)** Comparable infiltration of CD3^+^, CD4^+^, CD8^+^ TILs, and CD68^+^ CD163^+^ M2 tumor-associated macrophages between SCCCs and ACCs.

We compared proportions of CD3^+^, CD4^+^, and CD8^+^ T cells, CD57^+^ natural killer (NK) cells, CD4^+^ FOXP3^+^ regulatory T cells (T_regs_), and CD68^+^ CD163^+^ M2 tumor-associated macrophages (TAMs) between paired ACC and SCCC samples, respectively. In the total region, more T_regs_ (median proportion 0.63% vs. 0.58%, *p* = 0.029) and fewer NK cells (0.27% vs. 0.71%, *p* = 0.049) were enriched in SCCCs versus ACCs (**Figure 3A**), as subsequently evidenced by MIF results (**Figure 3B**). Consistently, these trends were detectable in tumorous and stromal regions (**Supplementary Figure 3**).

However, CD3^+^, CD4^+^, and CD8^+^ T cell proportions were comparable in SCCCs versus ACCs, either in the total region (**Figure 3C**) or tumorous and stromal regions (**Supplementary Figure 3**). M2 TAMs showed a similar trend between SCCC and ACC (**Figure 3C** and **Supplementary Figure 3**). Therefore, intratumoral heterogeneity of immune cell infiltration is present in lung ASC.

### Squamous cell carcinoma component reveals lower T-cell receptor diversity and higher T-cell receptor clonality than adenocarcinoma component

**Figure 4:**
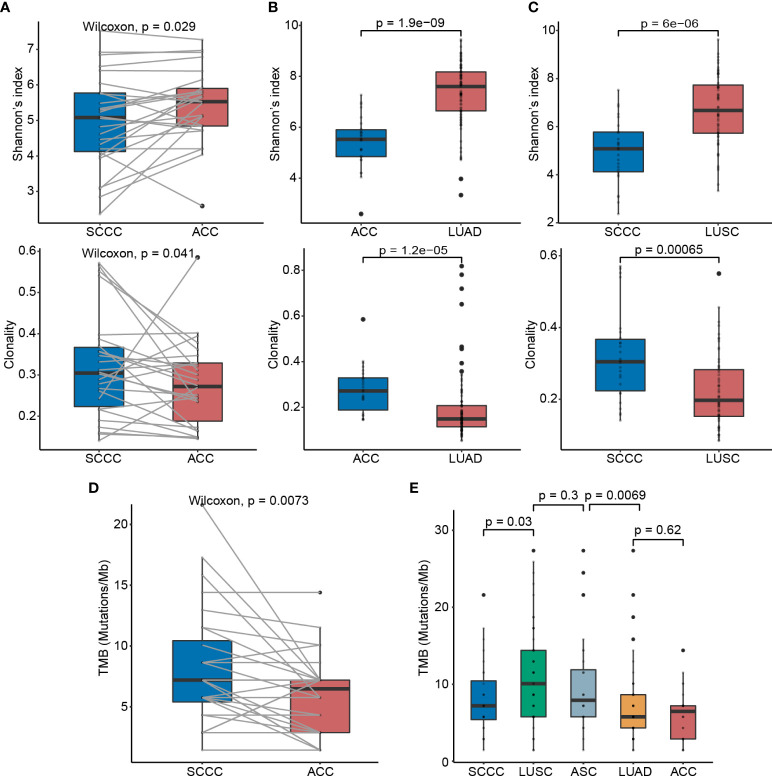
T-cell receptor (TCR) diversity and tumor mutation burden (TMB) status in lung adenosquamous carcinoma (ASC). **(A)** Comparisons of the TCR diversity and clonality between ACCs and SCCCs. Shannon’s index is employed to characterize tumor-resident T-cell diversity. TCR clonality reflects the potential clonal expansion of tumor-specific T cells. Lower TCR diversity and higher TCR clonality in **(B)** ACCs versus pure LUAD and **(C)** SCCCs versus pure LUSC. **(D)** TMB discrepancy between ACCs and SCCCs. **(E)** TMB comparisons among ACCs, LUAD, SCCCs, LUSC, and ASC.

We assessed the TCR repertoire diversity using TCRβ CDR3 sequencing and found that the TCR diversity in SCCCs was significantly lower than that in ACCs (median value 5.081 vs. 5.526, *p* = 0.029). The TCR clonality in SCCCs was higher than that achieved in ACCs (0.305 vs. 0.272, *p* = 0.041) (**Figure 4A**). Shannon’s index, as well as clonality, was proportional positively in ACC and SCCC. (*p* < 0.001 and *p* = 0.010) (**Supplementary Figures 2K, L**). These results suggested that the T cell diversity is still limited in SCCCs, together with boosted oligoclonal T-cell expansion, although the amount of CD3^+^ total T cells remains constant between the two types of pathological components.

Furthermore, we compared the TCR repertoire in ACCs versus stage-matched LUADs (*n* = 60), and in SCCCs versus stage-matched LUSC (*n* = 60), which were sequenced using the same platform and documented in Geneplus-Beijing. Still, SCCCs displayed lower TCR diversity and higher clonality than LUSC (both *p* < 0.001), and similar trends were found in ACCs versus LUAD (both *p* < 0.001) (**Figures 4B, C**). These findings demonstrated intratumoral heterogeneity of TCR repertoire between ACCs and SCCCs.

### Higher tumor mutation burden and intratumoral heterogeneity of immunogenicity in lung adenosquamous carcinoma

We sequenced the genomes of 28 pairs of ACCs and SCCCs to a median depth of 515×. The median TMB in SCCCs was 7.2 mutations/Mb (range 4 to 21.6), higher than that in ACCs (6.5 mutations/Mb, 1.4 to 14.4, *p* = 0.007) (**Figure 4D**), indicating the component heterogeneity of TMB within lung ASC resulted from the branch evolution between ACCs and SCCCs. The TMBs of ACCs and SCCCs were modestly proportional (*p* =0.002) (**Supplementary Figure 2**), influenced by component-shared mutations originated from the same genetic clone.

We next compared TMBs in the two pathological subtypes of lung ASC with those in pure, stage-matched LUADs (*n* = 170) and LUSCs (*n* = 62) (**Figure 4E**) obtained from Geneplus-Beijing. The median TMB in ACC was comparable with that in LUAD (4.3 mutations/Mb, 1.4 to 44.6, *p* = 0.620), whereas the SCCC TMB was lower than the archival LUSC (10.1 mutations/Mb, 1.4 to 27.4, *p* = 0.030). We further evaluated the median ASC TMB, which was calculated based on integration of non-synonymous somatic mutation (removing duplicates) data from ACC and SCCC samples. Lung ASCs showed a median TMB of 7.9 mutations/Mb (range 1.4 to 27.4), statistically higher than that in pure LUAD (*p* = 0.007) and close to that in LUSC (*p* = 0.300).

### Real-world outcome analysis of immune checkpoint inhibitor efficacy in lung adenosquamous carcinoma patients

Efficacy data from patients treated with anti–PD-1 agents (single or combined with chemotherapy agents) from 11 centers were retrospectively analyzed (**Tables 1**, **2**). Eighteen (39%) patients had complete PD-L1 expression information, and none of the patients had TMB records. Four *EGFR*-mutant patients were included.

**Table 1:** Demographic and disease characteristics of real-world lung ASC patients.

Characteristics	Total (*n*=46)	Mono-immunotherapy (*n*=20)	Chemoimmunotherapy (*n*=26)
Age			
Median (range), yr	60.5 (33-82)	60.5 (33-82)	60.5 (43-78)
<60 yr, no. (%)	21 (46)	9 (20)	12 (26%)
Sex			
Male, no. (%)	31 (67)	13 (65)	18 (69)
Female, no. (%)	15 (33)	7 (35)	8 (31)
Driver gene status			
*EGFR*, no. (%)	4 (9)	2 (10)	2 (8)
*ALK* fusion, no. (%)	1 (2)	0 (0)	1 (4)
*RET* fusion, no. (%)	1 (2)	0 (0)	1 (4)
*KRAS*, no. (%)	3 (7)	2 (10)	1 (4)
*MET* 14 skipping, no. (%)	1 (2)	0 (0)	1 (4)
Wild type, no. (%)	18 (39)	7 (35)	11 (42)
Unknown, no. (%)	18 (39)	7 (35)	11 (42)
Treatment line			
First, no. (%)	17 (37)	6 (30)	11 (42)
Second, no. (%)	14 (30)	9 (45)	5 (19)
≥Third, no. (%)	15 (33)	5 (25)	10 (38)

**Table 2:** Demographic and clinical information of real-world ASC patients.

Patient ID	Center	Sex	Age (yr)	Driver gene status	PD-L1 (TPS)	Treatment	Line	RECIST	PFS (months)	PFS status	OS(months)	OS status
rASC-01	Center 1	Male	67	Wild type	<1%	Immunotherapy+chemotherapy	1	SD	3.0	0	4.0	0
rASC-02	Center 1	Male	62	Wild type	NA	Immunotherapy+chemotherapy	≥3	PR	24.0	1	25.0	0
rASC-03	Center 2	Male	64	*EGFR* exon 19 deletion	<1%	Immunotherapy+chemotherapy	≥3	SD	5.4	1	18.4	0
rASC-04	Center 2	Male	61	Wild type	1%	Immunotherapy+chemotherapy	1	PD	1.6	1	13.4	1
rASC-05	Center 2	Female	61	*MET* exon 14 skipping	5%	Immunotherapy	≥3	PR	5.3	1	24.7	1
rASC-06	Center 2	Male	61	Wild type	40%	Immunotherapy	1	SD	11.5	0	11.5	0
rASC-07	Center 2	Male	74	Wild type	20%	Immunotherapy+chemotherapy	2	PR	15.6	0	15.6	0
rASC-08	Center 3	Male	77	*RET* fusion	NA	Immunotherapy	≥3	PD	1.3	1	10.7	1
rASC-09	Center 3	Male	64	Wild type	2%	Immunotherapy+chemotherapy	2	PD	2.5	1	2.6	0
rASC-10	Center 4	Male	63	NA	15%	Immunotherapy+chemotherapy	1	SD	20.9	0	20.9	0
rASC-11	Center 4	Male	68	*KRAS*	NA	Immunotherapy+chemotherapy	2	PD	0.7	1	16.4	1
rASC-12	Center 4	Female	47	Wild type	<1%	Immunotherapy+chemotherapy	1	PD	6.6	1	6.6	0
rASC-13	Center 4	Male	51	Wild type	NA	Immunotherapy	2	PR	1.4	1	1.4	1
rASC-14	Center 4	Male	43	Wild type	NA	Immunotherapy	2	SD	15.6	1	52.3	0
rASC-15	Center 4	Male	54	Wild type	NA	Immunotherapy+chemotherapy	1	SD	6.0	1	11.9	1
rASC-16	Center 4	Female	78	Wild type	NA	Immunotherapy	2	SD	24.0	1	44.9	0
rASC-17	Center 4	Male	60	Wild type	NA	Immunotherapy+chemotherapy	≥3	SD	7.9	1	22.7	0
rASC-18	Center 4	Male	57	KRAS	<1%	Immunotherapy	2	SD	30.2	0	30.2	0
rASC-19	Center 4	Male	68	Wild type	NA	Immunotherapy	1	SD	25.6	1	33.6	0
rASC-20	Center 5	Male	54	NA	NA	Immunotherapy	2	PD	2.6	1	6.0	1
rASC-21	Center 5	Female	50	NA	NA	Immunotherapy+chemotherapy	≥3	SD	1.9	1	12.7	0
rASC-22	Center 5	Female	60	NA	NA	Immunotherapy	2	PD	0.7	1	6.4	0
rASC-23	Center 5	Female	43	NA	NA	Immunotherapy+chemotherapy	1	SD	5.1	1	8.6	0
rASC-24	Center 5	Male	55	NA	NA	Immunotherapy	2	PD	1.3	1	15.4	1
rASC-25	Center 5	Male	82	NA	NA	Immunotherapy	1	PD	0.7	1	0.7	0
rASC-26	Center 6	Male	53	NA	≥50%	Immunotherapy+chemotherapy	≥3	SD	8.4	0	14.5	0
rASC-27	Center 6	Female	50	NA	NA	Immunotherapy+chemotherapy	≥3	PR	7.0	1	7.7	1
rASC-28	Center 6	Female	66	*EGFR* L858R+T790M	NA	Immunotherapy+chemotherapy	≥3	PR	18.8	0	26.1	0
rASC-29	Center 7	Male	59	NA	NA	Immunotherapy+chemotherapy	2	SD	6.4	0	6.4	0
rASC-30	Center 7	Male	48	NA	NA	Immunotherapy+chemotherapy	≥3	SD	3.3	1	18.7	0
rASC-31	Center 8	Female	68	NA	NA	Immunotherapy	2	PR	16.0	1	16.3	1
rASC-32	Center 8	Female	61	NA	NA	Immunotherapy+chemotherapy	1	PR	3.5	1	15.1	1
rASC-33	Center 8	Male	54	NA	NA	Immunotherapy	≥3	SD	2.0	0	2.0	0
rASC-34	Center 8	Female	51	NA	NA	Immunotherapy+chemotherapy	1	PR	8.0	1	10.7	0
rASC-35	Center 8	Male	63	NA	NA	Immunotherapy+chemotherapy	2	SD	11.2	1	11.2	0
rASC-36	Center 9	Male	55	Wild type	NA	Immunotherapy+chemotherapy	≥3	PD	1.5	1	1.5	0
rASC-37	Center 9	Female	68	*EGFR* L858R+T790M	NA	Immunotherapy	2	PD	0.7	1	1.5	1
rASC-38	Center 9	Male	52	NA	NA	Immunotherapy+chemotherapy	1	SD	5.0	1	6.7	0
rASC-39	Center 9	Female	61	*ALK* fusion	NA	Immunotherapy+chemotherapy	≥3	PD	1.5	1	1.5	1
rASC-40	Center 10	Female	53	Wild type	90%	Immunotherapy	1	PR	1.7	0	5.9	0
rASC-41	Center 10	Male	58	Wild type	15%	Immunotherapy+chemotherapy	1	PR	5.1	1	7.3	0
rASC-42	Center 10	Male	65	NA	70%	Immunotherapy	1	PR	9.8	1	36.1	1
rASC-43	Center 10	Female	33	*EGFR* R831H	60%	Immunotherapy	≥3	PD	0.7	1	0.7	0
rASC-44	Center 11	Male	62	*KRAS*	60%	Immunotherapy	1	PR	15.7	0	16.5	0
rASC-45	Center 11	Male	50	Wild type	≥50%	Immunotherapy	≥3	SD	6.0	1	7.1	1
rASC-46	Center 11	Male	78	Wild type	20%	Immunotherapy+chemotherapy	1	SD	6.8	1	7.0	0

**Figure 5:**
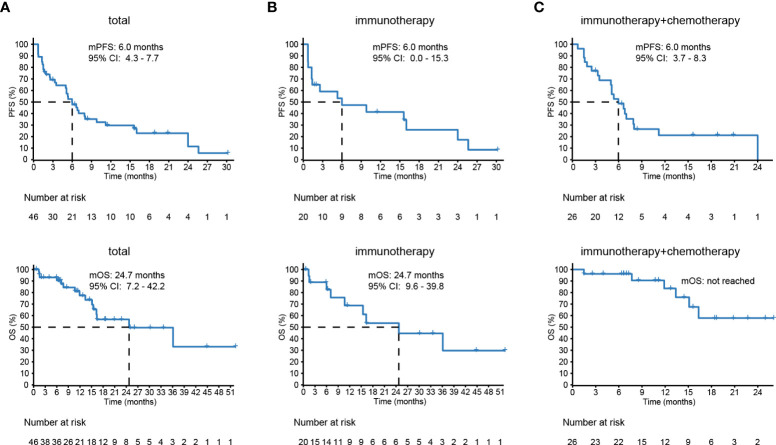
The survival of lung adenosquamous carcinoma (ASC) patients receiving immune checkpoint inhibitor (ICI)–based treatments. **(A)** Forty-six patients with advanced or recurrent lung ASC from eleven different centers show a median prognosis-free survival (PFS) of 6.0 months and overall survival (OS) of 24.7 months. **(B, C)** The PFS and OS of 20 patients treated with immunotherapy and 26 patients with chemoimmunotherapy.

Overall, 100% of patients underwent response evaluation per RECIST criterion. The proportions of patients achieving CR as best response, PR, SD, and PD were 0%, 28%, 43%, and 28%, respectively. These patients showed a median PFS of 6.0 months (95% CI 4.3–7.7 months) and OS of 24.7 months (95% CI 7.2–42.2 months) (**Figure 5A**). Twenty patients (43%) were treated with mono-ICIs, and 26 (57%) received ICIs plus standard chemotherapy. In the mono-ICI subgroup, patients achieved an ORR of 30% (CR, *n* = 0 and PR, *n* = 6) and a DCR of 65% (PR/SD, *n* = 13) with median PFS and OS of 6.0 (95% CI 0.0–15.3) and 24.7 (95% CI 9.6–39.8) months (**Figure 5B**), compared with a comparable ORR of 27% (PR, *n* = 7) and a higher DCR of 77% (PR/SD, *n* = 20) in chemo-ICI patients, with median PFS of 6.0 (95% CI 3.7–8.3) months. However, during the median follow-up of 12.2 months, OS was not evaluated due to the special endpoint result—only six patients were deceased (**Figure 5C**).

Among four *EGFR*-mutant patients, two of them treated with mono-ICIs both achieved PD within 1 month; another two patients treated with chemo-ICIs were controlled, with 5-month PFS and 18-month OS. Among seven PD-L1–positive patients treated with mono-ICIs, four achieved PR, two achieved SD, and one achieved PD. Their median PFS was 6.8 (95% CI 2.4–11.2) months, and OS was unevaluable. Among another seven PD-L1–positive patients treated with chemo-ICIs, two achieved PR, three achieved SD, and two achieved PD, with median PFS and OS of 9.8 (95% CI 4.8–14.8) and less than 13.1 (95% CI 0–50.3) months.

## Discussion

Lung ASC patients are still refractory to antitumor treatment, particularly those with no actionable mutations. In this study, we ascertain that the TIME in lung ASC is characterized by immune-excluded or immune-desert phenotype and shows intratumoral heterogeneity regarding immune cell infiltration, immune checkpoint expression, TMB, and TCR repertoire between ACCs and SCCCs. For far too long, previous studies of immunotherapy efficacy in lung ASC have never reported a larger real-world cohort than our analysis, which shows a moderately satisfactory response.

Recently, with a large gene panel and whole-exome sequencing, shared mutations in ACCs and SCCCs have been shown to result in the monoclonal origin of lung ASC (11, 21). These shared alterations are trunks in the phylogenetic tree and are, particularly actionable mutations (e.g., *EGFR* and *KRAS* mutations), frequently identified in driver genes, underlying the proposal of simultaneous treatment for pathologically biphasic components. *EGFR* mutations have been proven to have strong associations with immunotherapy resistance in NSCLC (22). *MDM2* amplification, considered a factor leading to hyperprogression during immunotherapy in NSCLC (23), is also frequently detected in lung ASC (11). These high-frequency resistance-related genomic factors should therefore be elicited during the management of lung ASC.

The immunophenotypes that we detected may confer characteristics of “altered” and “cold” tumors in lung ASC, a new approach for tumor classification (hot, altered, and cold immune tumors) proposed by Camus *et al.* for primary colorectal cancer in 2009 (24). It is generally believed that hot tumors can efficiently respond to immunotherapy, whereas altered tumors have an inadequate response, and cold tumors have no response to immunotherapy. However, the gross classification using hot or cold is insufficient to characterize an intricate TIME in some refractory tumors. Altered tumors represent an intermediate phenotype. An international study previously predicted recurrence and survival risks in patients with three tumor subtypes (25). In this study, we associate immune-inflamed, immune-excluded, and immune-desert immuno-phenotypes with hot, altered, and cold tumors. The reason for this redefinition is immunophenotyping based on TIL density in tumorous and stromal regions showed that lung ASC was not regularly hot tumors as the total amount of TILs in the tumorous region was lower than 10%, and that lung ASC had relatively higher immunogenicity as it had a relatively higher TMB than LUAD but was comparable with that in LUSC. These characteristics indicate that the use of immunotherapy to improve the outcome is challenging, although higher TMB has been utilized as a predictor for monitoring ICI efficacy or adjustment of the initial strategy (26). Our finding that lung ASC patients receiving mono-immunotherapy and chemoimmunotherapy had an ORR of 28% and PFS of 6.0 months marks a benefit of these patients from ICI-based therapy, although ASC has been categorized into altered and cold tumors.

Molecular and cellular heterogeneity between ACCs and SCCCs has been demonstrated in this study, which can be associated with branch evolution and selection. SCCCs had higher PD-L1 expression, TMB, TCR clonality, and T_regs_ proportions than ACCs. Particularly, PD-L1 was predominantly expressed in SCCCs compared with ACC. Consistently, a recent study also reported that PD-L1 expression discrepancy between the two histological components within lung ASC could be seen on images, although the histological data were not compared (27). PD-L1 expression in squamous cells was also observed in 15 pancreatic ASCs (28). PD-L1 has been identified as an immune checkpoint molecule contributing to immune evasion. Our previous study showed that SCCCs of lung ASCs could be transformed from ACC (11), similar to LUSC transformation from LUAD that has been observed in several cases after EGFR-TKI treatment (29). From these results, it can be hypothesized that the selective PD-L1 expression may encourage SCCC branch evolution through the suppressed regulation of the immune microenvironment. Lung ASC also has a significant association with a poor prognosis (4–6). Immune escape associated with high PD-L1 expression in SCCCs can be partly explained by less inflammatory infiltration to tumor cells in this component. However, we are unable to reveal the genetic mechanism for selective PD-L1 expression and SCCC histogenesis due to incomplete gene panel sequencing information.

The limitations are apparent, including limited sample size and the selected population in which most cases are driver gene-negative in the real-world cohort. This selection bias is associated with sparingly clinical attempts at immunotherapy in *EGFR*-mutant ASC patients, as *EGFR*-mutant NSCLC is generally considered to be less responsive to immunotherapy. Unfortunately, we failed to directly analyze the association between TIME and treatment efficacy in the real-world cohort, which is expected to be explored in future prospective studies (30, 31).

## Conclusions

Lung ASC features high-frequent *EGFR* mutations, generally suppressive TIME, and genetic and immuno-heterogeneity between ACCs and SCCCs. Lung ASC patients without *EGFR* mutations have a moderate response to ICI-based immunotherapy.

## Data availability statement

The data presented in the study are deposited in the NCBI Sequence Read Archive, accession number PRJNA865459.

## Ethics statement

The studies involving human participants were reviewed and approved by Fujian Cancer Hospital Ethics Committee. The patients/participants provided their written informed consent to participate in this study.

## Author contributions

GL, BZ, and CL conceived the study idea and designed the study. CL and XBZ prepared research materials. HJW, JH, LW, ZJW, HG, FW, WZZ, CZZ, QC, JZ, LFZ, KJ, QM, YQX, SWW, HBW, SSY, YJL, and CH collected clinical data. WJX and WFZ performed pathology. PSL, XLZ, and QL performed guidance to them for accurate bioinformatics and statistical analyses. GL was accountable for all aspectsof the study. CL, XBZ, PSL drafted the manuscript or revised it critically for important intellectual content. All the authors reviewed the paper and approved the final manuscript.

## Funding

This study was supported by the China Southwest Oncology Group (CSWOG), a national collaborative clinical research group of 97 member hospitals, National Natural Science Foundation of China (82072565), Beijing Xisike Clinical Oncology Research Foundation (Y-2019AZZD-0386), Fujian Provincial Health and Family Research Talent Training Program[2018- CX-12], Fujian Provincial Science and Technology Department guided projects (2020Y9038), Fujian Provincial Health Systemic Innovation Project (2020CXA010), Fujian Provincial Health Commission (2020QNA014), Fujian Provincial Natural Science Foundation (2020J011120), Fujian Provincial Natural Science Foundation (2021J01432), Youth Program of National Natural Science Foundation of China (82102991), Fujian Provincial Natural Science Foundation (2021J05084), and Fujian Provincial Clinlical Research Center for Cancer Radiotherapy and Immunotherapy (2020Y2012). Fujian Key Laboratory of Advanced Technology for Cancer Screening and Early Diagnosis.

## Conflict of interest

Authors PL, QL, XX and XY were employed by the company Geneplus-Beijing.

The remaining authors declare that the research was conducted in the absence of any commercial or financial relationships that could be construed as a potential conflict of interest.

## Publisher’s note

All claims expressed in this article are solely those of the authors and do not necessarily represent those of their affiliated organizations, or those of the publisher, the editors and the reviewers. Any product that may be evaluated in this article, or claim that may be made by its manufacturer, is not guaranteed or endorsed by the publisher.
