# Supplementary material for: Heterogeneity of tumor immune microenvironment and real-world analysis of immunotherapy efficacy in lung adenosquamous carcinoma
Source: Front Immunol. 2022 Aug 12;13:944812. doi: 10.3389/fimmu.2022.944812 (PMC9413057; doi:10.3389/fimmu.2022.944812)
Supplement: Supplementary file 1 [file DataSheet_1.docx]

**Supplementary Materials**

**
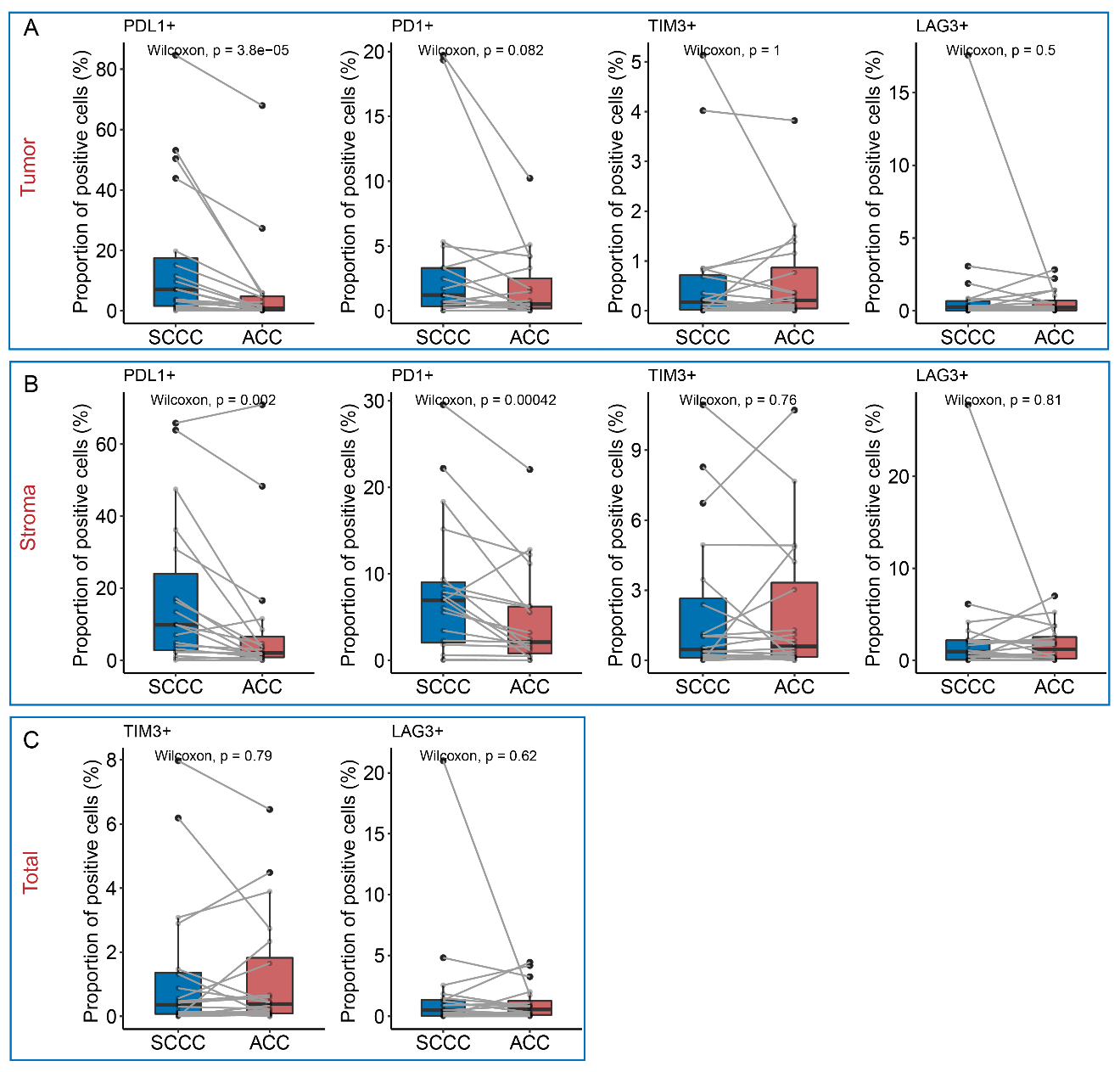
**

**Supplementary Figure 1** The density of immune cells expressing immune checkpoints in tumorous, stromal, and total regions using multiple immunofluorescences.

**
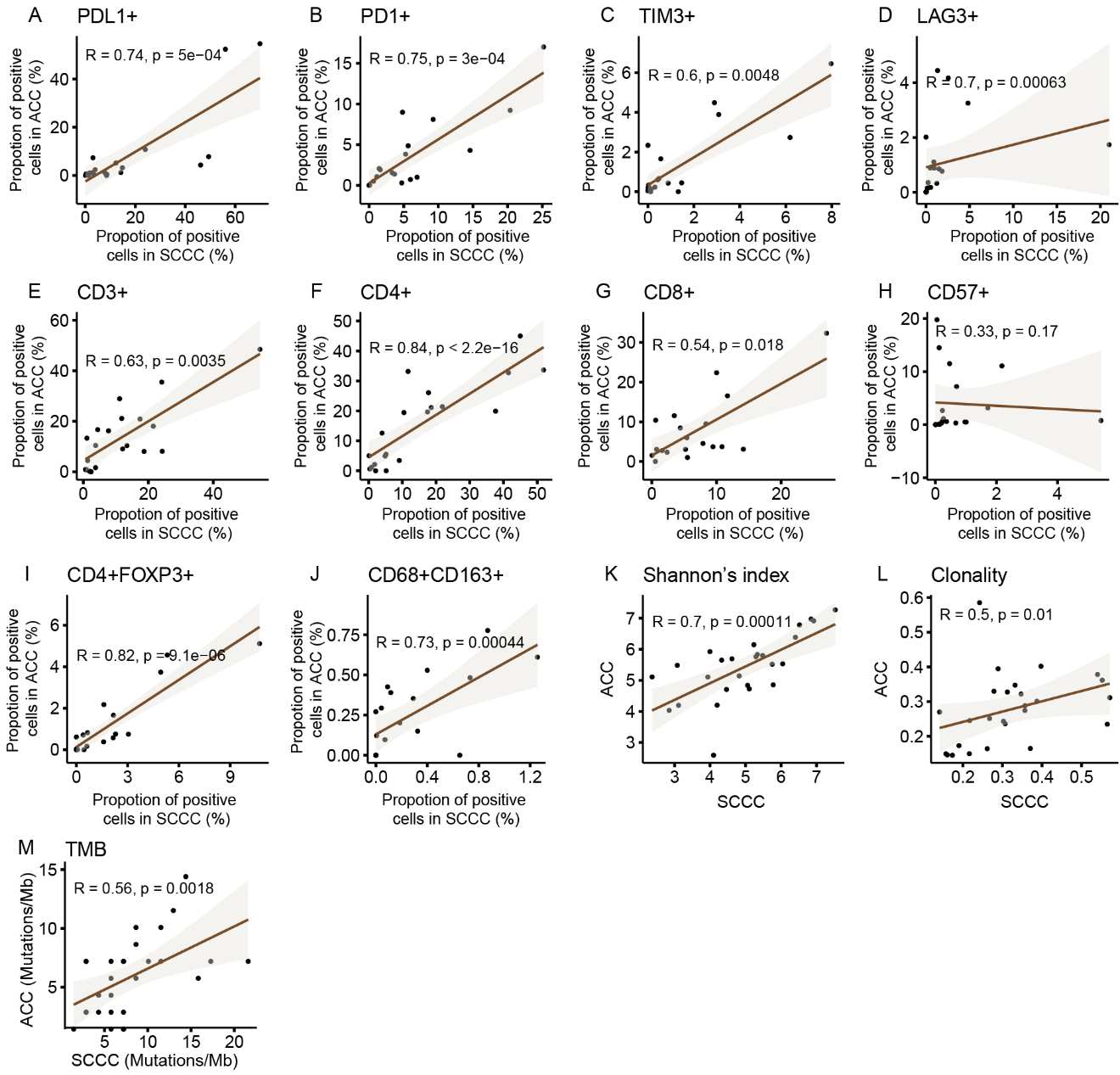
**

**Supplementary Figure 2** Correlation analyses of immune checkpoints, TMB, TILs, and TCR with the proportions of ACC and SCCC.

**
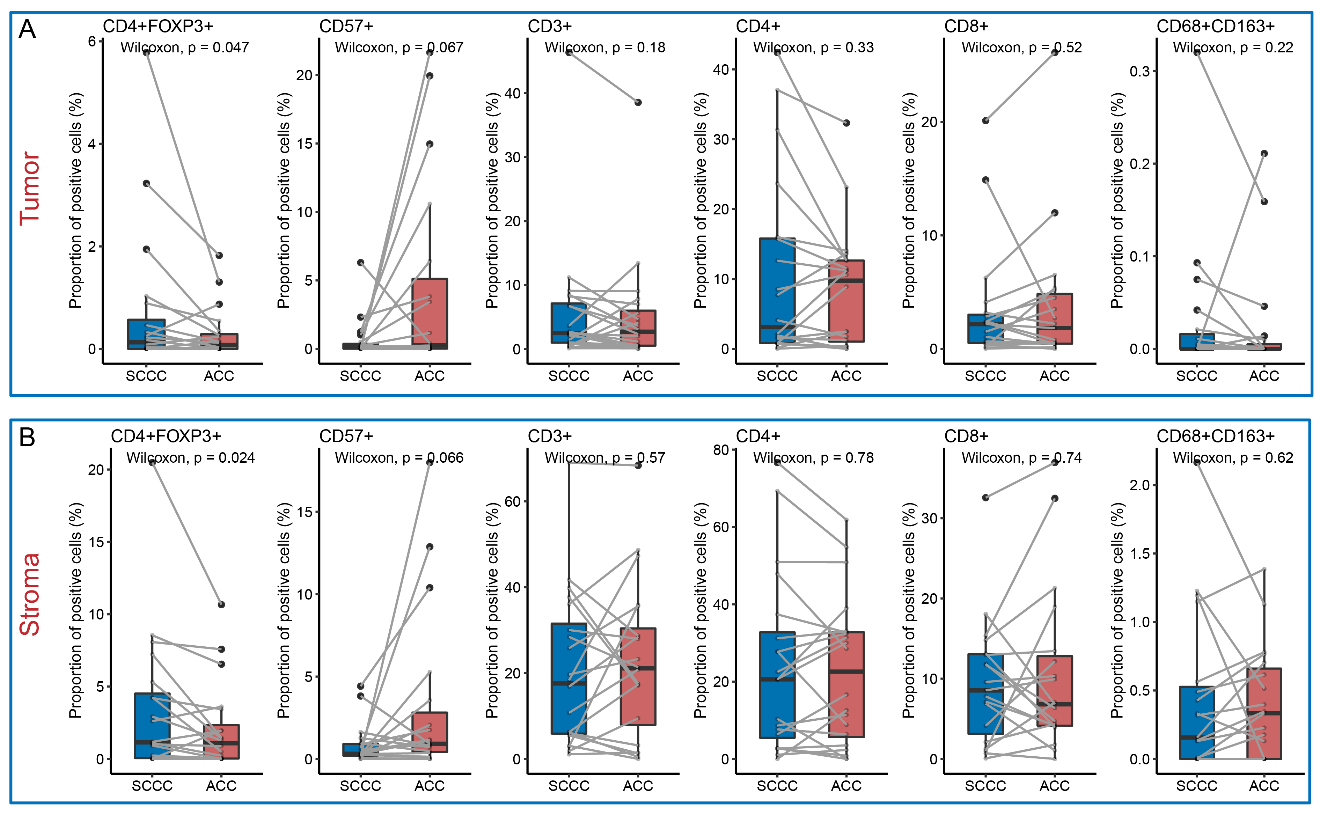
**

**Supplementary Figure 3** Comparisons of immune cell density between SCCC and ACC in tumorous and stromal regions. Immune cell subpopulations comprise CD3^+^ T cells, CD3^+^ 4 cells, CD4^+^ FOXP3^+^ Tregs, CD57^+^ NK cells, and CD68^+^ CD163^+^ M2 tumor-associated macrophages.
